# Integrated Geoscientific Surveys at the Church of Santa Maria della Lizza (Alezio, Italy)

**DOI:** 10.3390/s21062205

**Published:** 2021-03-21

**Authors:** Lara De Giorgi, Ivan Ferrari, Francesco Giuri, Giovanni Leucci, Giuseppe Scardozzi

**Affiliations:** Institute of Heritage Science, National Research Council of Italy, 73100 Lecce, Italy; lara.degiorgi@cnr.it (L.D.G.); ivan.ferrari@cnr.it (I.F.); francesco.giuri@cnr.it (F.G.); giuseppe.scardozzi@cnr.it (G.S.)

**Keywords:** GPR, laser scanner, Santa Maria dell Lizza church, Alezio (Italy)

## Abstract

The church of Santa Maria della Lizza is one of the most important examples of medieval architecture in the Salento Peninsula in south Italy. In order to understand the extension and layout of the crypts, integrated ground-penetrating radar (GPR) prospections and laser scanner surveys were undertaken in the church and in the surrounding areas. The analysis of the GPR measurements revealed many anomalies that could be ascribed to unknown structures (crypts), as well as other anomalies related to the old church. The GPR data were supported by the laser scanner data establishing the spatial relationship between the surface and the level below the church.

## 1. Introduction

**Figure 1:**
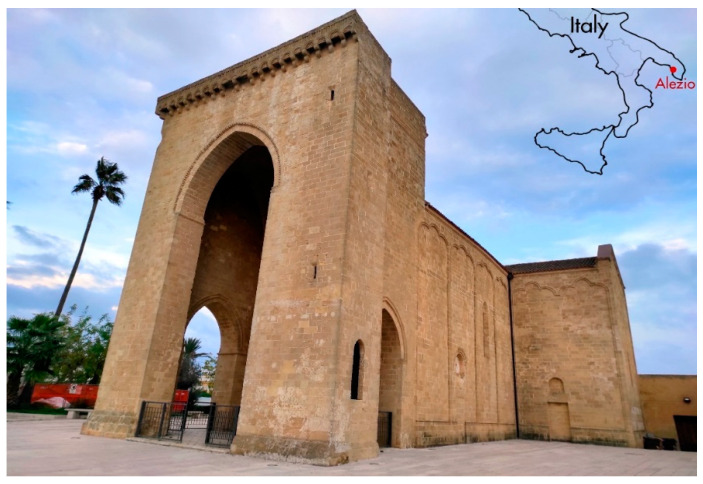
Church of Santa Maria della Lizza in Alezio (Lecce, Italy). South-west view of the high turreted pronaos.

**Figure 2:**
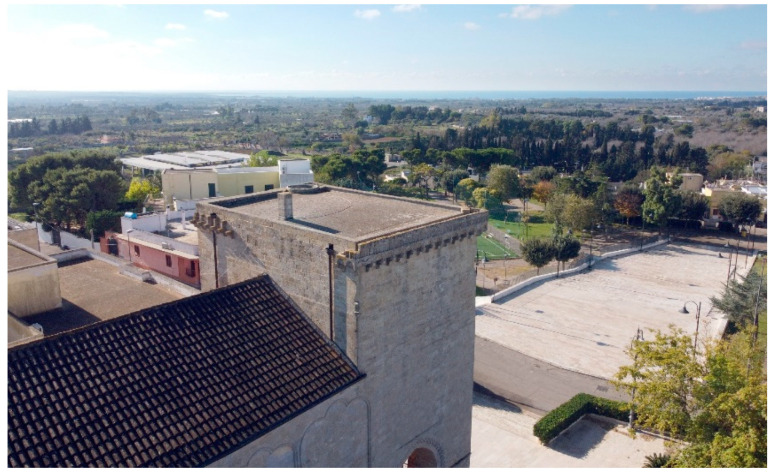
Church of Santa Maria della Lizza in Alezio (Lecce, Italy). Aerial view from the north-east.

The church of Santa Maria della Lizza in Alezio (Figure 1) is a very important example of late medieval religious architecture in the Apulia region in southern Italy. Located on the western side of the Salento peninsula about 6.5 km east of Gallipoli, with its high turreted pronaos it dominates a vast territory projected onto the Ionian Sea (Figure 2). The building is linked to the ancient cult of the Assumption of Mary, defined as “della Lizza” or “di Alizza” because it was built on the homonymous hill, characterized by a toponym that derives from the ancient Messapian-Roman settlement of Aletium, in which the Lizza hill was one of the most important sectors [1].

**Figure 3:**
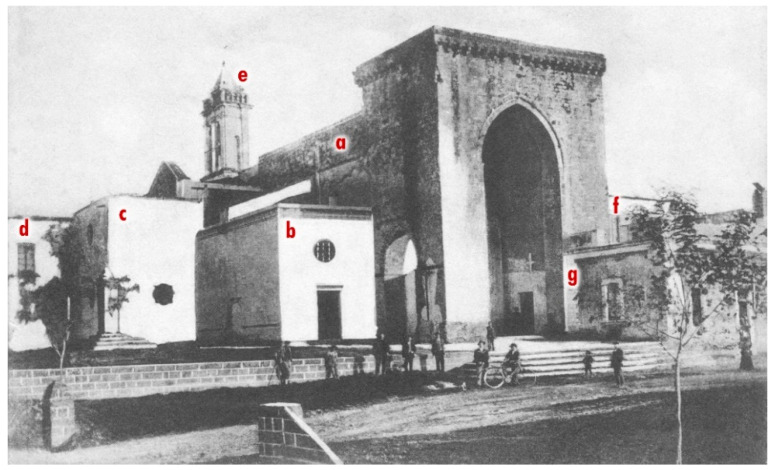
Church of Santa Maria della Lizza in Alezio (Lecce, Italy). Photographic document from the early 20th century that still shows: the masonry vaults (**a**), the small northern nave (**b**), the sacristy (**c**), the bishop’s palace (**d**), the bell tower (**e**), the Congrega’s house (**f**) and buildings leaning against the pronaos (**g**) (after [6]).

The church underwent various construction phases, which over time have strongly altered its appearance. The original layout is presumably datable to between the end of the 13th and the beginning of the 14th century. It was characterized by a Latin cross plan outlined given by the intersection of the nave and the apsed presbytery (about 30 × 10 m) with a monumental strongly projecting transept (about 21 × 8 m), externally embellished with raised pilasters which, branching off from the plinth at the bottom, rejoin curving upwards to form elegant trefoil arches [2,3]. More properly assignable to the 14th century is the construction of the monumental turreted pronaos (18 m high) leaning against the entrance of the church, open with pointed arches on three sides and vaulted with a ribbed cruise. This is a rarity in the panorama of medieval architecture of the Italian peninsula [4]. Starting from the 16th century and up to the 19th century at the behest of various bishops of Gallipoli, the church was the subject of heavy building interventions that transformed the austere and slender medieval appearance internally embellished with colorful frescoes [5], into a building of worship with typically baroque connotations. The roof with wooden trusses was replaced by cross vaults in masonry supported by mighty pillars leaning against the walls of the nave and transept, which together with the rich and majestic Baroque altars obliterated the ancient frescoes. The old single-lancet windows were bricked up and new ones were made by forcibly cutting into the walls. A new nave was built on the northern front adjacent to the central one and connected with two large and high arched openings in the walls, plus a third to the east that gave access to the northern branch of the transept. The room used as a sacristy was set against the north front of the transept, while the bishop’s palace occupied the corner between the transept and the presbytery, the point where the bell tower also stood; on the southern external front of the pronaos and aisle and up to the transept was the *Cappellone* of the Congrega (Figure 3). Probably as a result of all these interventions, towards the first half of the 20th century the church began to show more and more evident signs of structural failure, which lead to the impressive and questionable restorations of the years 1959–1961. In this circumstance, a conservative intervention was not pursued, but a stylistic restoration that eliminated all the baroque evidence to give the building its original appearance again.

In this way the trusses were restored, in the presbytery the pointed arch with the upper choir and the apse behind it was rebuilt, the pointed arches at the intersection between the nave and the transept were also rebuilt, the walls in the damaged portions were repaired, reopened the original doors and windows and finally brought to light the surviving frescoes. These restoration works involved the internal demolition of vaults, pillars, altars and any other sacred furnishings, while externally, with the exception of the episcope, the Congrega’s house, the small northern nave, the old sacristy and the bell tower were also demolished, plus further buildings built at the turn of the 19th and 20th centuries close to the pronaos [7]. It was during these works that the rooms of the underground cemetery below the church were discovered, due to the subsidence of the floor in the north arm of the transept. It consists of a series of small rooms, independent from each other, built in square blocks of local stone and barrel vaulted, whose only access points were quadrangular entrances of 60/80 cm side, placed in the floor of the church and closed by removable tombstones: through this opening alone the dead were lowered into the underground rooms for their burial. In the first centuries after Christ, the practice of burials *ad sanctos* or *martyribus sociatus* was instituted to facilitate the path of the deceased towards the resurrection. During the Medieval and post-Medieval times, the nobles were deposited in underground rooms located near or in front of family chapels; the clergymen, on the other hand, were buried in a single underground room, located near the presbytery and the main altar; the commoners were piled without a coffin, wrapped only in a shroud, and deposited in several underground corridors under the main nave. This type of hypogeum cemetery had an important tradition in Christian Salento (as for example document the important cases in the cathedrals of Lecce and Nardò) and was adopted until the early 19th century, when after the Napoleonic edict of Saint Cloud for hygienic and sanitary reasons, burial in urban centers was forbidden to relegate it to special extra-urban areas. Despite this, the Aletine community continued to bury their dead in the Lizza underground cemetery until the second half of the 19th century.

This contribution aims to illustrate the results achieved by the joint research activity of the Information Technologies Laboratory (ITLab) and the Laboratory of Applied Geophysics of the Institute of Heritage Sciences (ISPC) of the CNR of Lecce (south Italy), in the context of a wider scientific collaboration project stipulated with the Municipality of Alezio (Lecce Province) and the Salento University, with the cooperation of the Archaeological Superintendence. The investigations involved the Church of Santa Maria della Lizza and more specifically the underground rooms located below the building and the surrounding area. Although the discovery of the underground rooms dates back to about sixty years ago, a plan of the hypogeum cemetery has never been published. However, it is an essential element to understand (i) the real extension and distribution of the underground rooms and their relationship with the structures of the church, and (ii) the dimensions of the individual compartments and the spatial relationship between them. Therefore, according to a working methodology already adopted in other research activities performed in cooperation by the two laboratories [8,9,10], in some cases also conducted on the same territory [11], laser scanning surveys and ground-penetrating radar (GPR) investigations were carried out to fill the gap in the knowledge of this context.

The laser scanning surveys were aimed at producing a 3D model of the church, including the external churchyard and the underground rooms. Some graphic tables have been created, which have been very useful in the examination of some technical-construction details of the monument, especially linked to the different construction phases. Furthermore, the survey was preparatory to the planning of the GPR prospecting campaign that has allowed for the identification of structures unknown until now, both inside and outside the church, fundamental to offer a more organic knowledge and understanding of the transformations of a monument still little studied. 

## 2. Materials and Methods

### 2.1. Laser Scanner Survey 

Laser scanner survey was performed using a Leica P20 instrument ((Leica, Wetzlar, Germany). The coverage of the entire underground area was achieved by planning a total of 40 station points, including part of the external area facing the two underground entrances for a precise connection to the church structures: due to the small size of the rooms, but above all due to the absence of relevant decorative details, it was set a resolution of 12.5 mm on a sphere of 10 m of radius, quality three. The measurement resolution chosen made it possible to have an easily manageable amount of data considering the numerous points of stations planned at regular intervals for the total coverage of the area. For processing data has been used a workstation equipped with an Intel Xenon ES-2630 2.30 GHz processor, 32 GB of RAM and an Nvidia K 4000 graphics card. The acquired data were first processed in Cyclone software (v 8.1.1, Leica, Wetzlar, Germany) to align the point clouds and to export them in a single .pts file of 11 GB with 352 million points. Subsequently the points were decimated in Geomagic (v Studio 2003, Morrisville, NA, USA) to get a polygonal mesh with a good level of detail easy to manage: the 3D model of 4.1 million of polygons was exported in .obj file of about 200 MB.

**Figure 4:**
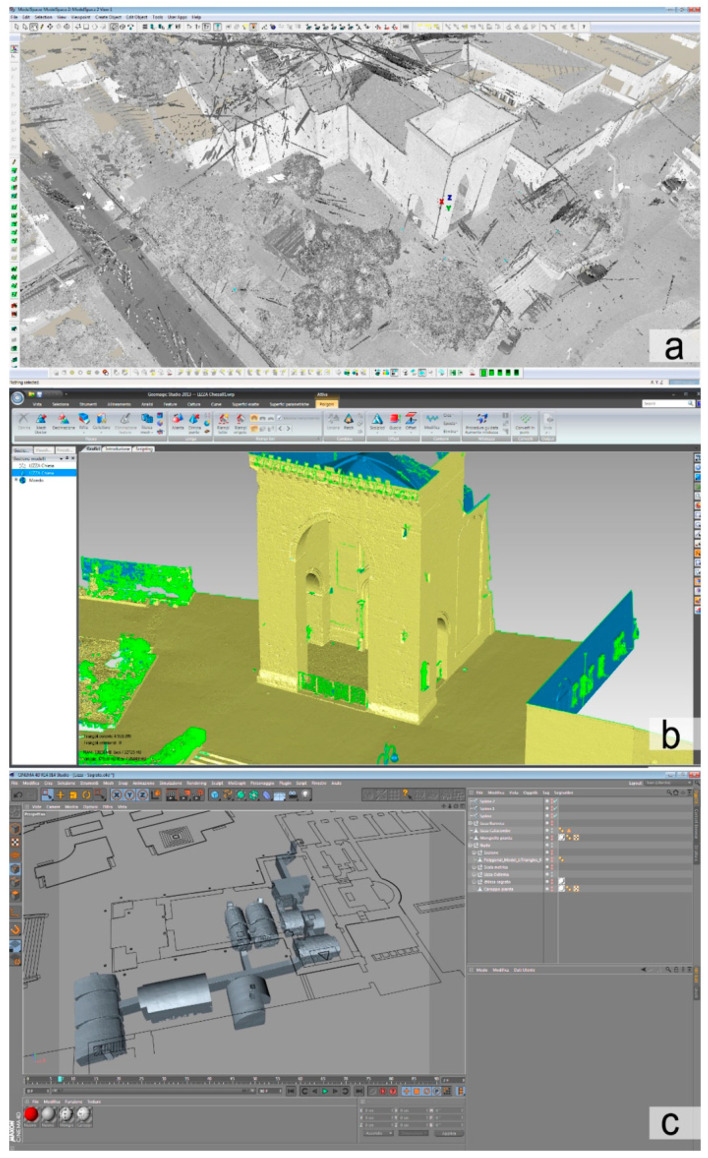
Screenshot of the survey campaign data processing: (**a**) the 1.3 billion point cloud; (**b**) the polygonal surface processed from the point cloud; (**c**) the model optimization and the creation of plans, sections and renderings.

The same workflow was used for a 3D laser scanner survey of the church and the external churchyard. To cover the entire area, 78 additional station points were necessary, and to ensure a good degree of accuracy of the high portions it was adopted an instrument resolution of 6.3 mm on a sphere of 10 m radius. The exported .pts file of 32.4 GB has about 1.3 billion points; from it a 3D .obj model of 590 MB of about 65 million polygons was processed, and it is divided into eight parts of 5 million polygons each for the church and a further 5 parts of 5 million each for the churchyard. All these models were finally imported and merged into Maxon C4D (v R14.014) for the creation of plans, sections and rendering (Figure 4).

### 2.2. GPR Data Acquisition

**Figure 5:**
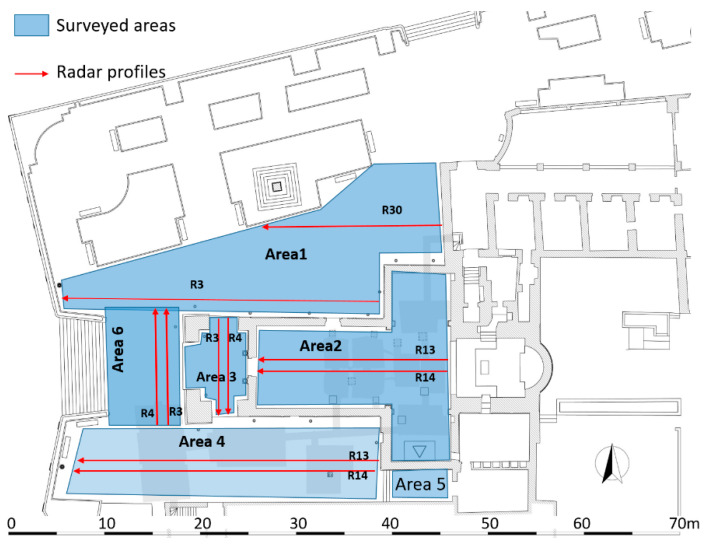
The GPR surveyed areas with location of some profiles.

A GPR survey was performed on six areas labelled Area 1-Area 6, respectively (Figure 5): Area 1 is an open space immediately to the north of the church, corresponding to the demolished northern nave and old sacristy (see Section 2 above); Area 2 is inside the church, including the transept and the nave; Area 3 is inside the pronaos; Area 4 is an open space immediately south of the pronaos and the nave, corresponding to the demolished Congrega’s house (see above paragraph 2); Area 5 is a small open space south of the transept; Area 6 is an open space in front of the pronaos, partially corresponding with the northern sector of the demolished housing (see above paragraph 2). For the GPR measurements the georadar Ris Hi-Mod was used. with the aim of obtaining a good compromise between resolution and depth of investigation, the dual band antenna was used with central band frequencies equal to 200 and 600 MHz. GPR data were acquired with parallel profiles spaced 25 cm. Successively data were processed by the follow steps: (i) zero timing; (ii) background removal filter [12]; (iii) gain variable versus depth [13]; (iv) Kirchoff migration [13]. For this last step the electromagnetic wave propagation velocity of 0.11 m/ns, evaluated from diffraction hyperbolas [13], was used.

The GPR-slice software [14] was used. The GPR data analysis show that only the data obtained from the 600 MHz antenna were significative and therefore only the data obtained from 600 MHz will be analyzed here. After appropriate data processing, various three-dimensional visualization techniques are currently used to display the GPR data in a convenient way for understanding the spatial relationships between the anomalous zones and improving their archaeological interpretation. The most popular GPR visualization method is as time-slice (or depth-slice) maps [15,16,17] since it provides a comprehensive plan view of the anomaly patterns at various depths, easy to correlate with structures within increasingly deeper archaeological units. This is a useful that creates maps of reflected wave amplitude differences in discreet horizontal time slices within a grid [18,19,20,21,22,23]. This type of visualization plays an important role in unravelling the complex layers within urban settings and the evolution of buildings and urban landscapes through time [18,19,24,25,26]. Amplitude slice-maps were constructed using the overlay analysis [17]. This allow to the strongest and weakest reflectors at the depth of each slice are assigned specific colours and therefore allow to see the subtle features that are indistinguishable on radargrams.

## 3. Results

### 3.1. Laser Scanner

The plan obtained by instrumental survey was compared with the only one published to date [7]. Differences emerged both in the metric accuracy and in the alignment of the structures. For this reason, an attempt was made to combine the instrumental survey with the old technical drawing showing the plans of the demolished buildings, this not considering the whole of the drawing, but analyzing separately those of the individual buildings to minimize the percentage of relative error.

**Figure 6:**
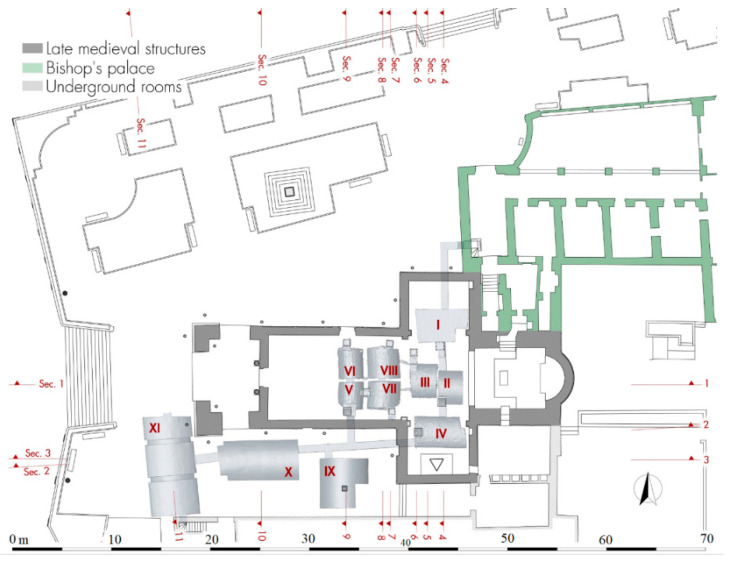
General plan, from laser scanner survey, showing the underground rooms documented by laser scanner and the section lines of Figure 10.

By analyzing the 3D model of the underground sector, it is possible to clearly understand the size of the individual rooms, their location, the levels and the path of the modern corridors created during the 1959–1960 restorations to provide a tourist visit route. The underground rooms are accessible by a staircase placed in the entrance hall of the bishop’s palace, which leads into a passage 70 cm wide and 2.60 m height. The corridor, after a first stretch to west direction, turns 90° to the south, passes the foundations in the north wall of the transept and lead to the first series of four funerary cells placed below the transept (Figure 6, I–IV). 

**Figure 7:**
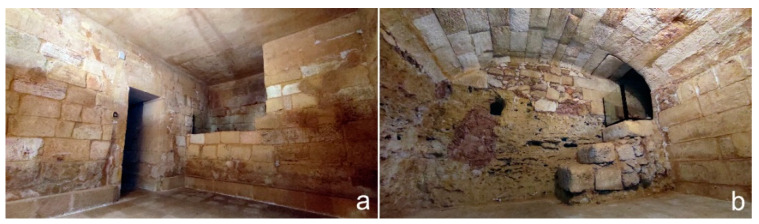
Underground rooms: (**a**) sepulchral cell I; (**b**) sepulchral cell III.

**Figure 8:**
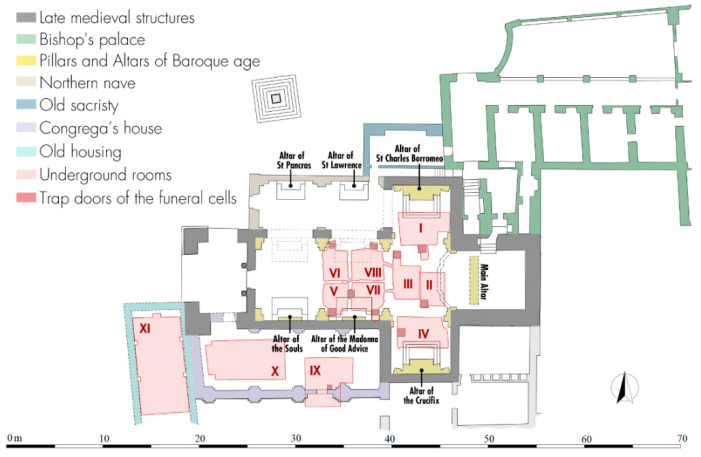
The new general plan laser scanning survey integrated with the plans of the buildings demolished during the 1959–1961 restorations from Mongiello 1974.

The lateral rooms I and IV are larger, have an almost specular polygonal plan and originally both had a depressed barrel vault with an east-west axis: today the northern one has a flat concrete modern roof built in the 1959–1961 restoration due the collapse of the church floor in this point (Figure 7a). The trap door in room I located on the southern side is sealed, unlike that of room IV placed on the western side and protected by a metal grate: this is one of the three trap doors opened during the restoration for the Great Jubilee in 2000, when the Lizza sanctuary was celebrated its jubilee stage. By comparing the current data with those of the church in the Baroque period, we understand how the presence of the altars of St. Charles Borromeo on the north and the Crucifix on the south have led the cells to be built away from the back walls of the transept. It also understood how the particular plan shape was linked to the presence of the pillars supporting the cross vaults and the need to avoid gaps undermining the stability of the building (Figure 8).

The two smaller central rooms (II and III) are almost aligned with each other on an axis, have a rectangular plan and depressed barrel vaults with an east-west axis. The trap door of room III located in the north-west corner is sealed (Figure 7b), while the one in room II located in the south-west corner is open and protected by a grate: this last room was the most important one by its position in front of the main altar, here the clerical exponents were buried.

The two smaller central rooms (II and III) are almost aligned on an axis with each other, have a rectangular plan and depressed barrel vaults with an east-west axis. The trap door of room III located in the north-west corner is sealed (Figure 7b), while the one in room II located in the south-west corner is open and protected by a grate: this last room was the most important one by its position in front of the main altar, here the clerical exponents were buried.

**Figure 9:**
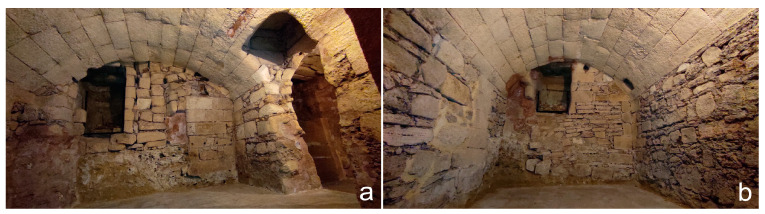
Underground rooms: (**a**) the sepulchral cell VI; (**b**) the sepulchral cell VII.

From the room IV a new corridor moves toward west through an opening in the foundations of the transept wall, and with a short staircase it is possible to descend further 1.2 m. After six meters, a branch of the visit path turns 90° northwards and oversteps the southern wall of the nave: here is a second group of further four cells (Figure 6, V–VIII) reaching by going up four steps and having all a rectangular plan with a depressed barrel vault on the north-south axis. The southern rooms (V and VII) have their own trap doors on their southern sides plus a third in the middle communicating with both: among these, the western one is the third that was reopened and protected by a metal grate in 2000 (Figure 9a). The couple of northern rooms (VI and VIII) have instead their respective trap doors each on the northern side (Figure 9b). Also in this case, by superimposing the current plan on that of the Baroque building, we see how the three southern trap doors touch the three sides of the podium of the demolished altar of the Madonna of Good Advice. Reversing this observation on the two northern hatches, it is easy to suppose an identical and specular situation relating to an altar no longer existing in the 20th century, but present before the construction of the small northern nave (Figure 8).

**Figure 10:**
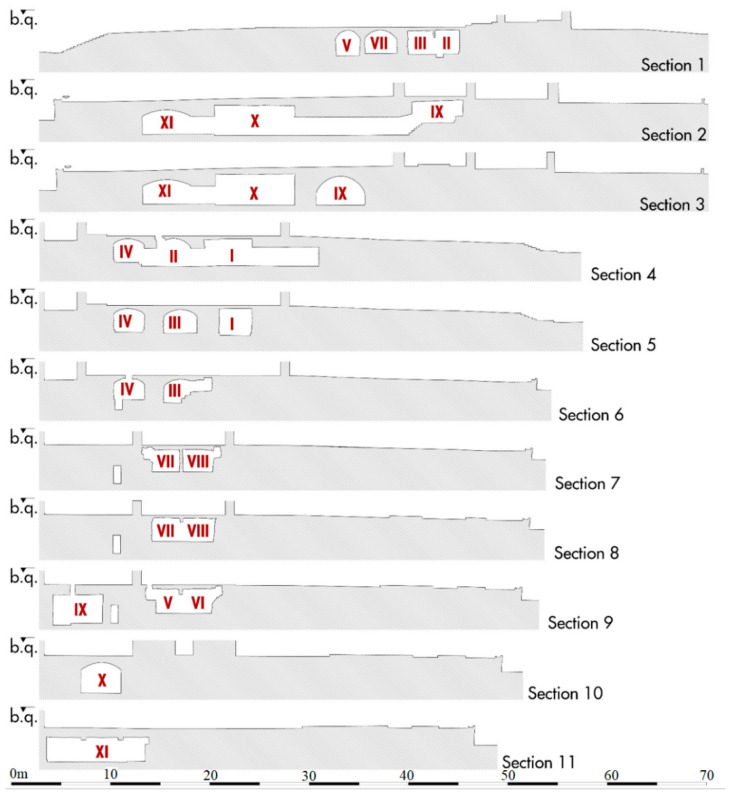
Sections of the underground rooms from laser scanner survey (general plan Figure 6).

Analyzing the different sections of the 3D model, we can see how the floors of the eight funeral cells are at a depth between −2.60 and −2.85 m from the floor of the church. The vaults reach a thickness of only 25 cm at the highest points (Figure 10). Small ventilation ducts in the walls allowed for the circulation of gases from the decomposition of corpses and their expulsion outside for air exchange.

**Figure 11:**
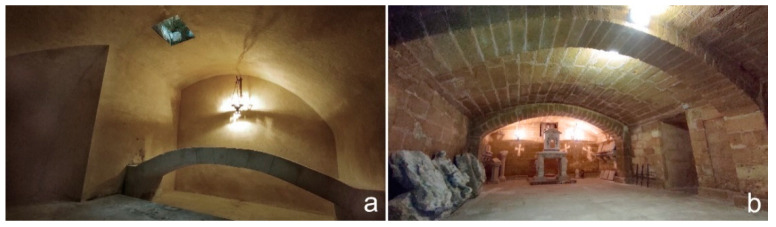
Underground rooms: (**a**) the cistern (IX); (**b**) the food warehouse (XI).

Coming back to the east-west corridor, a passage on the south side lead to a large L-shaped cistern covered by a depressed barrel vault with a north-south axis (Figure 5, IX), which probably pre-existed the construction of the Congrega’s house because of partly extended below its southern wall (Figure 11a). 

**Figure 12:**
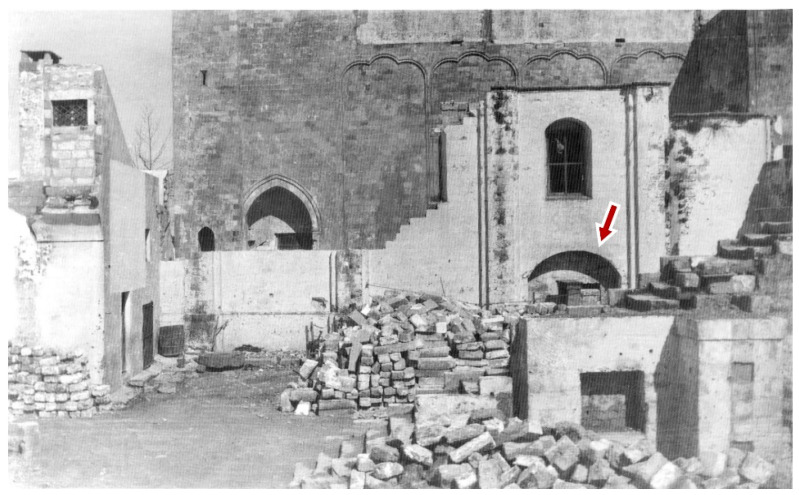
Photographic document from 1960 showing the Congrega’s house demolishment: the red arrow points to the arch above the cistern.

Precisely for this reason, it is likely that the cistern originally had a square plan, which was then modified to give a masonry base to the building’s pillar putted in the south-east corner (Figure 8). A further element that emerged from a rare photographic document taken during the demolition of the building is the presence of a large arch in the southern wall of the Congrega’s house right above the cistern: a technical solution that allowed the weight of the structure to unload not directly on its vault, but rather laterally to it (Figure 12). The mouth of the tank for gathering rain waters has a quadrangular shape (50 × 50 cm); it is along the median axis near the cistern on southern side, exactly placed in line with the internal curtain wall of the demolished building.

The penultimate room reached by the modern corridor was the large sepulchral cell reserved for members of the Confraternity of the Lizza, previously positioned under the Congrega’s house (Figure 6, X). It is a rectangular room (8.10 × 4.00 m) characterized by a depressed barrel vault with an east-west axis, whose modern plastering no longer allows to understand where the trap door was placed: the slightly oblique profile of the western side would tend to outline the layout of the back wall of the same Congrega’s house.

The last and largest room has a rectangular north-south oriented plan (10.00 × 4.90 m) and a depressed barrel vault reinforced by two arches 50 cm wide in the middle part (Figure 6, XI). The longitudinal axis is parallel to the western side of the Congrega’s sepulchral cell and it is likely that the houses once close to the pronaos had the same alignment, in order to not unload the weight on the vault (Figure 11b). The memoirs describe this underground place as cool and dry and used for the storage of grain and other provisions needed by the religious during their summer holidays in Alezio.

Finally, a staircase with a U-path goes from the southern side of room XI up to the external churchyard near the southern side of the high pronaos.

### 3.2. GPR

**Figure 13:**
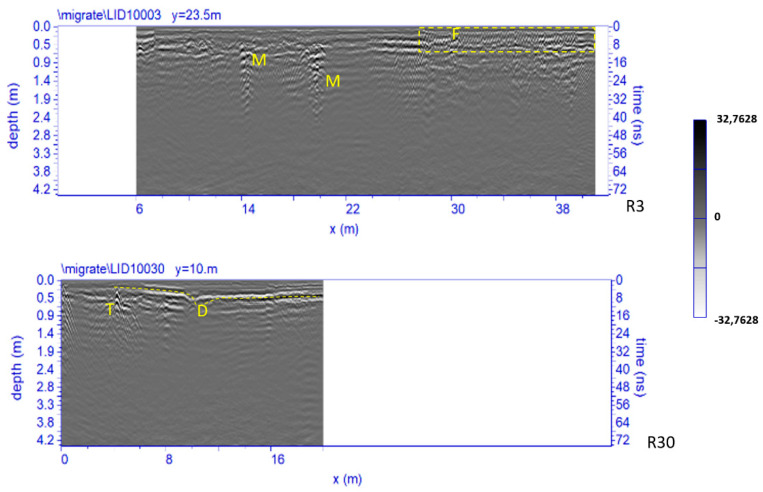
The GPR processed radar sections related to the Area 1; D: partial collapse event; F: metal reinforcement; M: walls; T: pipes.

Figure 13 shows two processed radar sections related to Area 1. Four significant anomalies, labelled D, F, M and D are clearly visible. The anomaly F, inside the dashed yellow box, has a typical shape of small hyperbolas probably due to the presence of a metal reinforcement (electro-welded mesh) of the paving; the anomalies M at a depth between 0.5 m and 1.0 m are probably related to the presence of walls; anomaly T at a depth between 0.2 m and 0.3 m is probably related to the presence of pipes.

A partial collapse event is indicated with D. This event could have been due to the presence of small voids possibly caused by rainy events and probably aggravated by the passage of heavy vehicles. 

**Figure 14:**
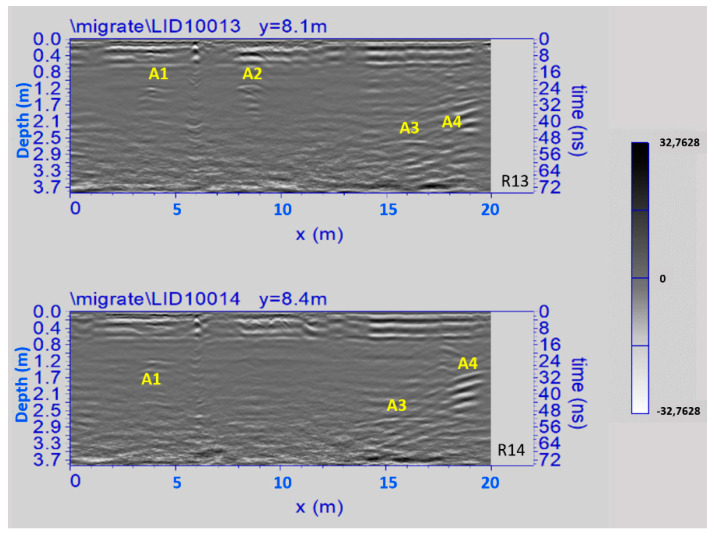
The GPR processed radar sections related to the Area 2; A1 and A2: known parts of the underground rooms; A3 and A4: unknown underground rooms.

Figure 14 shows two processed radar sections related to Area 2. Four significant anomalies, labelled A1, A2, A3 and A4 are clearly visible. The anomalies A1 and A2, at depth between 0.5 m and 1.0 m, are probably related to the presence of the already known parts of the underground rooms II, III, VI and VIII (see above paragraph 3); the anomalies A3 and A4, at depth between 0.8 m and 2.5 m are probably related to the presence of one or two unknown underground rooms located west of room VI. 

**Figure 15:**
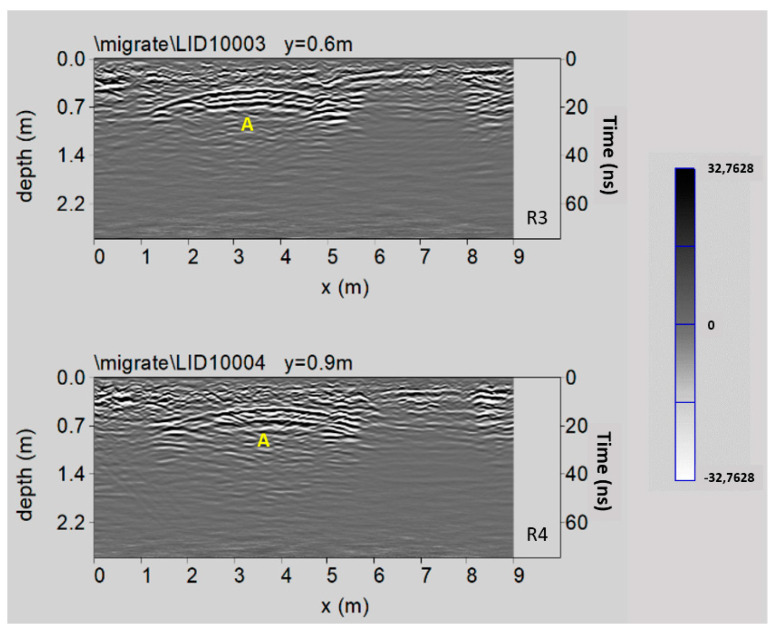
The GPR processed radar sections related to the Area 3; A: empty underground room.

Figure 15 shows two processed radar sections related to Area 3. An interesting reflected event is highlighted (labelled A) at a depth between 0.5 m and 0.7 m. This event could be due to the presence of an empty underground room, which was subsequently filled. 

**Figure 16:**
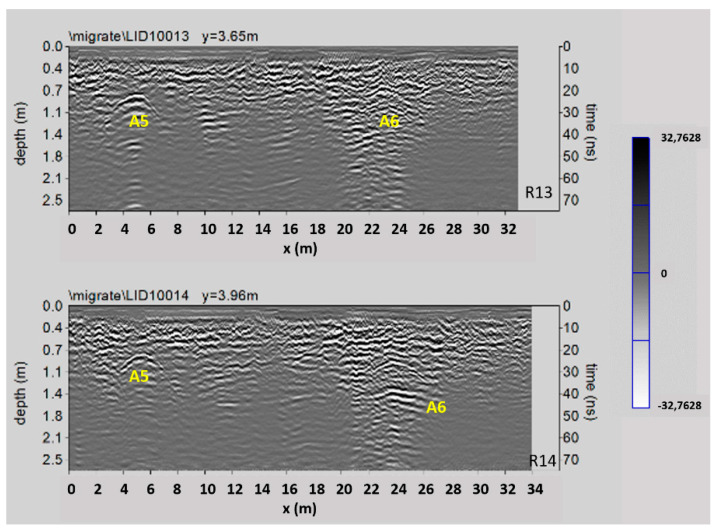
The GPR processed radar sections related to the Area 4; A5 and A6: known underground rooms.

Figure 16 shows two processed radar sections related to Area 4. Here the reflected events at a depth between 0.5 m and 1.0 m (indicated with A5 and A6) are visible. They are probably relating to the presence of the already known parts of the underground rooms IX and X (see Section 3 above). 

**Figure 17:**
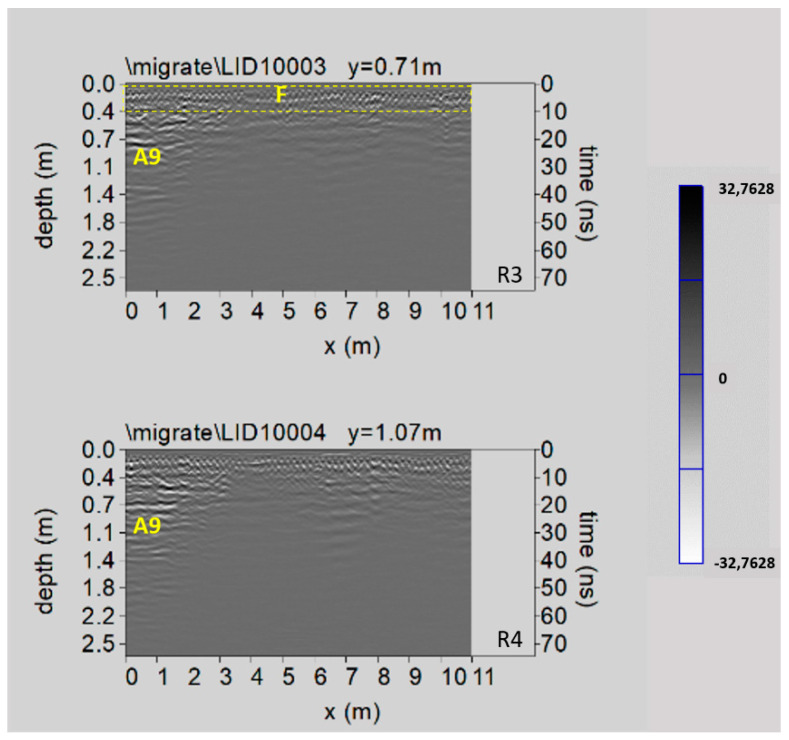
The GPR processed radar sections related to the Area 6; F: metal reinforcement; A9: known crypt.

Figure 17 shows two processed radar sections related to Area 6. Here some reflected events, indicated by F within the dashed yellow box, are related to the presence of a metal reinforcement (electro-welded mesh) of the paving; other reflected events at depth between 0.7 m and 1.1 m (labelled A9) probably related to the presence of the northern part of the already known crypt XI (see above paragraph 2).

In the very small Area 5 no significant results were found. 

The time slices were built using a time interval Δt = 5 ns that correspond to a thickness of 27.5 cm. One approach for visualizing 3D is the use of iso-surface rendering, assembled from all processed radargrams [18,19,20,27,28].

**Figure 18:**
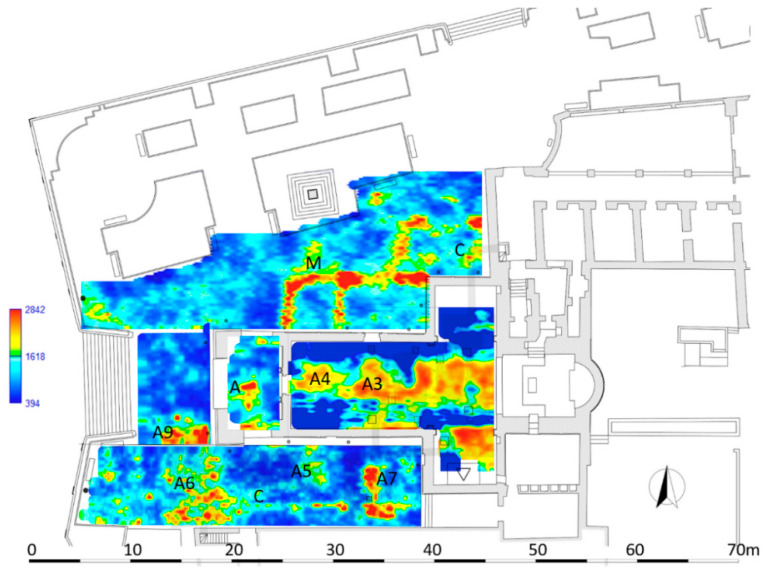
The GPR time slices: Area 1 (0.7–0.9 m depth); Area 2 (0.8–1.0 m depth); Area 3 (0.4–0.5 m depth); Area 4 (0.8–0.9 m depth); Area 6 (0.5–0.7 m depth).

Figure 18 shows the most significant time slices superimposed on the plan of the areas investigated. The time slices show the normalized amplitude using a range defined by blue as zero and red as 1.

In the time slices ranging from 0.7 m to 0.9 m depth related to the Area 1 (Figure 18), relatively high-amplitude alignments (labelled M, respectively) are clearly visible. These correspond to the anomalies labelled M in the radargrams (Figure 13). These anomalies are related to the walls relative to the old structure of the church demolished in 1959–1961 (specifically the northern nave). Another anomaly, labelled C, is visible in the same time slice. This anomaly is related to the well-known entrance corridor to the underground system of crypts.

In the time slice related to the Area 2 (Figure 18) ranging from 0.8 m to 1.0 m depth the anomalies labelled A3 and A4 are well evidenced. They are related to the unknown develop of the system of crypts below the surface of the church. Specifically, they could be related to one or two unknown underground rooms located west of the room VI documented by the laser scanner survey (Figure 8). In the easternmost part of this Area 2, some anomalies could be also linked to the demolished structures of the old sacristy. 

In the time slice related to the Area 3 (Figure 18), ranging from 0.4 m to 0.5 m depth, the anomaly labelled A evidences the presence of an unknown filled cavity located under the paving of the pronaos. 

In the time slice related to the Area 4 (Figure 18), ranging from 0.8 m to 0.9 m depth, the anomalies A5, A6 and A7 confirm the well-known sector of the system of crypts; specifically, the anomalies A5 and A6 correspond to the rooms IX and X, while anomaly A7 is related to the well-known corridor that connects these rooms to the other crypts. Furthermore, the anomaly labelled C is visible in the same time slice and I related to the southern wall of the demolished Congrega’s house. 

Lastly, in the time slice related to the Area 6 (Figure 18), ranging from 0.5 m to 0.7 m depth, the anomaly A9 confirm the well-known northern part of the crypt XI.

## 4. Discussion

**Figure 19:**
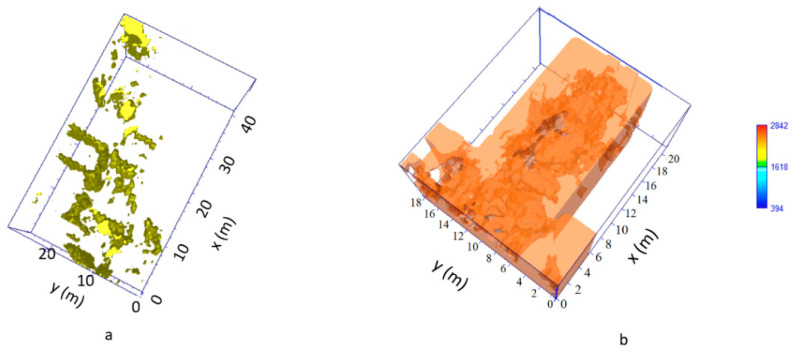
The GPR iso-surfaces: (**a**) Area 1; (**b**) Area 2.

In order to improve the interpretation of geophysical anomalies, GPR data were integrated with the 2D and 3D models produced by laser scanner survey. In particular, GPR time slices and profiles were georeferenced in the map of the church. First of all, the GPR data interpretation was favoured by the iso-amplitude surface visualization (Figure 19). The GPR results highlighted a series of anomalies regarding various buried archaeological structures. Among the anomalies evidenced in the area 1 can be identified as the remains of the old built structure of the church (Figure 13, M). Other anomalies with high amplitude are due to the presence of unknown buried rooms (Figure 14, A3 and A4). In the other investigated areas numerous anomalies corresponding to known buried rooms. Particularly in the area 3 (Figure 15, A) a filled buried room was found. Traces of this room had been lost. At this point is important to integrate the GPR data with the laser scanner data. This step is fundamental to place the structures into a three-dimensional framework.

**Figure 20:**
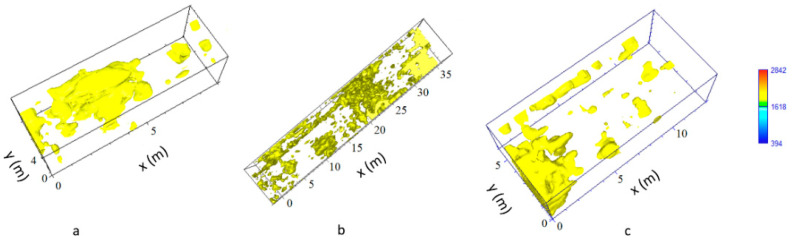
The GPR iso-surfaces: (**a**) Area 3; (**b**) Area 4; (**c**) Area 6.

**Figure 21:**
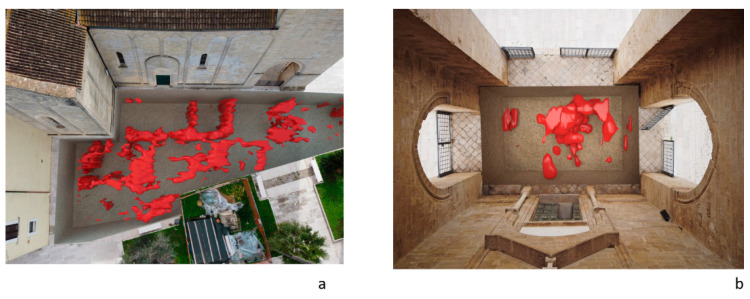
The 3D representation of the underground anomalies in the Areas 1 (**a**) and 4 (**b**).

The created iso-surfaces volume (Figure 19 and Figure 20) was exported in a 3D ascii files in order to be merged with the polygonal surfaces obtained with the laser scanner data. Here is possible to insert the GPR iso-surfaces volume at the exact depth. In this way a sort of virtual excavation is obtained in which the georadar anomalies are positioned in the exact spatial position (Figure 21a,b) and allow to compare the known structures with those detected by the geophysical data allowing to get a relationship between the visible and the not visible. 

## 5. Conclusions

Thanks to the integration with laser scanner data, GPR survey offered new data on the extension of underground rooms and buried walls.

In particular, the laser scanner survey has allowed for the production of the complete map of the known crypts. In this new plan it was possible to distinguish the exact position in space (shape and depth) of the various crypts. The geophysical prospecting allowed us to highlight numerous anomalies, many of them of archaeological interest, in part related to the known crypts (rooms II-XI) and ancient structures demolished in 1959–1961 (such as the northern nave and the Congrega’s house), and in part linked to new underground rooms or cavities (such as anomalies A, A3 and A4, in the nave of the church and in the pronaos), previously unknown and yet to be explored.

The integration with the laser scanner data allowed us to validate the results of GPR survey. Indeed, it was possible to compare the events reflected of the electromagnetic waves on known underground structures with those of unknown buried structures on the radar section and allow their interpretation. Moreover, the georeferenced time slices allow the identification of the exact position of the unknown buried rooms. The 3D iso-surfaces inserted into the virtual environments facilitate the interpretation phase and allowed us to increase the knowledge of the studied monument evidencing its past and present structure organization. The results will also be useful for the planning of future targeted excavations.

## Data Availability

The data presented in this study are available on request from the corresponding author.
